# Quality Assessment of Clinical Practice Guidelines on the Treatment of Hepatocellular Carcinoma or Metastatic Liver Cancer

**DOI:** 10.1371/journal.pone.0103939

**Published:** 2014-08-08

**Authors:** Yingqiang Wang, Qianqian Luo, Youping Li, Haiqing Wang, Shaolin Deng, Shiyou Wei, Xianglian Li

**Affiliations:** 1 The Chinese Evidence-based Medicine center/The Chinese Cochrane Centre, West China Hospital, Sichuan University, Chengdu, China; 2 Department of Medical Administration, 363 Hospital, Chengdu, China; 3 National Chengdu Center for Safety Evaluation of Drugs, West China Hospital, Sichuan University, Chengdu, China; 4 Institute of Preventive Medicine, Yichun University, Yichun, China; 5 West China Medical School, West China Hospital, Sichuan University, Chengdu, China; Copenhagen University Hospital, Denmark

## Abstract

**Objectives:**

To assess the quality of the currently available clinical practice guidelines (CPGs) for hepatocellular carcinoma, and provide a reference for clinicians in selecting the best available clinical protocols.

**Methods:**

The databases of PubMed, MEDLINE, Web of Science, Chinese Biomedical Literature database (CBM), China National Knowledge Infrastructure (CNKI), WanFang, and relevant CPGs websites were systematically searched through March 2014. CPGs quality was appraised using the Appraisal of Guidelines for Research & Evaluation (AGREE) II instrument, and data analysis was performed using SPSS 13.0 software.

**Results:**

A total of 20 evidence-based and 20 expert consensus-based guidelines were included. The mean percentage of the domain scores were: scope and purpose 83% (95% confidence interval (CI), 81% to 86%), clarity of presentation 79% (95% CI, 73% to 86%), stakeholder involvement 39% (95% CI, 30% to 49%), editorial independence 58% (95% CI, 52% to 64%), rigor of development 39% (95% CI, 31% to 46%), and applicability 16% (95% CI, 10% to 23%). Evidence-based guidelines were superior to those established by consensus for the domains of rigor of development (p<0.001), clarity of presentation (p = 0.01) and applicability (p = 0.021).

**Conclusions:**

The overall methodological quality of CPGs for hepatocellular carcinoma and metastatic liver cancer is moderate, with poor applicability and potential conflict of interest issues. The evidence-based guidelines has become mainstream for high quality CPGs development; however, there is still need to further increase the transparency and quality of evidence rating, as well as the recommendation process, and to address potential conflict of interest.

## Introduction

Hepatocellular carcinoma (HCC) is the seventh most common cancer worldwide [1], and the third most common cause of death from cancer with an overall mortality-to-incidence ratio of 0.93[2]. Most of the burden is in developing countries, where almost 85% of cases occur [1], [2]. The annual cost of HCC in the United States is $454.9 million, with an average cost per patient of $32,907. Healthcare costs and lost productivity account for 89.2% and 10.8% of the total, respectively [3]. A survey showed that the cost for patients with HCC is approximately 6 to 8 fold higher than for those without this cancer, with the mean per-patient-per-month (PPPM) cost of $7,863 for cases and $1,243 for controls [4]. It is estimated that the number of disability-adjusted life years (DALYs) lost and medical costs due to HCC will gradually increase as the incidence of HCC rises in younger people.

The Institute of Medicine (IOM) has established the definition of clinical practice guidelines (CPGs) as “systematically developed statements to assist practitioner and patient decisions about appropriate health care for specific clinical circumstances” [5]. This will provide doctors with detailed and authoritative recommendations and alter their customary or outdated clinical methods, which will improve healthcare consistency, promote health service equity and reduce healthcare costs for the government [6]. Currently, although the quantity and quality of CPGs have been improved, the differences among guidelines formulated by various institutes or researchers still differ widely. Therefore, a rigorous evaluation of the quality of CPGs is urgently needed. Appraisal of Guidelines for Research & Evaluation (AGREE II) is recognized as a preferred tool for the quality appraisal of guidelines [7], [8]. This can provide a methodological strategy for the development of guidelines, and inform authors on the type of information and the manner in which the information should be reported in the guidelines, thereby ultimately improving the level of healthcare [9].

Schmidt *et al* [10] evaluated the quality of 32 guidelines on the diagnosis and treatment of HCC in 2011. They concluded that most guidelines lacked appropriate methodological quality. However, all guidelines they included were published before 2010 and were assessed using the original four-point scale of the AGREE instrument published in 2003, which is not in compliance with current methodological standards of health measurement design. In particular, this noncompliance might threaten the performance and reliability of the instrument [8]. The aim of the present study is to systematically assess the quality of current available CPGs for HCC or metastatic liver cancer using the AGREE II instrument, and provide a reference for clinicians in selecting the best clinical protocols.

## Materials and Methods

### Inclusion criteria

The available guidelines on the treatment of primary or metastatic liver cancer published in English or Chinese were included.

### Exclusion criteria

a) HCC guidelines for diagnosis (i.e., ultrasound, enhanced computerized tomography (CT)); b) The Chinese version or other versions of oversea CPGs; c) Quality improvement guidelines, position statements or guideline summaries; d) National Institute for Health and Excellence interventional procedure guidance (NICE IPG) or overview; e) Conference abstracts, overviews, primary studies, systematic reviews or letters.

### Guideline sources and search strategy

The electronic databases of PubMed, MEDLINE, Web of Science, Chinese Biomedical Literature database (CBM), China National Knowledge Infrastructure (CNKI), and WanFang were systematically searched through March 2014. The MeSH terms with free-text terms were as follows: (Liver Neoplasms OR Carcinoma, Hepatocellular) AND (Guideline OR Practice Guideline OR Consensus). We also searched the relevant CPG websites, including Guideline-International Network (G-I-N), National Guideline Clearinghouse (NGC), Clinical Practice Guideline Network (CPGN), National electronic Library for Medicines (NeLM), and NICE.

### Selection of Guidelines

The PRISMA (preferred reporting items for systematic reviews and meta-analyses) statement was followed to search and select guidelines [11]. Two reviewers (WYQ, WSY) independently screened guidelines by browsing title and abstract based on predefined inclusion and exclusion criteria. Primary screening of the guidelines was undertaken by two reviewers who carefully read the full text to determine their eligibility for inclusion in the study. Discrepancies between the two reviewers were resolved by discussion or with a third person (LYP).

If a guideline has clearly stated the quality of evidence on which a recommendation is based or grading for recommendation and statements, then the guideline is judged as evidence-based. If a guideline is developed based on consensus (i.e., consensus meeting or expert panel), without illustrating the source of evidence and grade of recommendation, the guideline is judged as consensus-based.

### Quality appraisal

Three appraisers (WYQ, WSY and WHQ) independently rated the included CPGs using the AGREE II instrument that consisted of 23 key items organized within six quality domains followed by two global rating items (“Overall Assessment”). Each of the items was rated on a 7-point scale (1-strongly disagree to 7-strongly agree). The appraisers scored each guideline independently using the rating scale. If the three appraisers rated items with a difference of more than two points, a consensus discussion was held to obtain the final rating [10]. Observed scores of individual items in a domain were calculated by summing up all scores of the three appraisers, and each domain score was standardized as a percentage according to the following formula [9]:
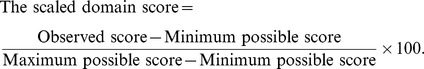



[Maximum possible score = 7 (strongly agree) × No. of items within a domain × No. of appraisers; Minimum possible score = 1 (strongly disagree) × No. of items within a domain × No. of appraisers].

A domain score of 60% was considered a threshold value of the AGREE instrument for rating the overall quality of CPGs. A guideline was ‘strongly recommended’ if the majority of domains (more than five) were scored above 60%. A guideline was ‘weakly recommended’ if more than four domains were scored above 30%. A guideline was ‘not recommended’ if more than three domains were scored below 30% [10].

### Statistical analysis

The mean score and 95% confident intervals (CI) were calculated for each domain using AGREE II. Kendall's coefficient of concordance [12] was applied for estimating the reliability among appraisers. The independent sample Student's t-test was applied if a result of Levene's test was p>0.05. Data and graphics were performed using SPSS version 13.0 for Windows (LEAD Technologies, Inc., IL, USA) and SigmaPlot version 12.0 for Windows (Systat Software, Inc., Chicago, IL), respectively. A p-value of less than 0.05 was considered significant.

## Results

### Search results

**Figure 1:**
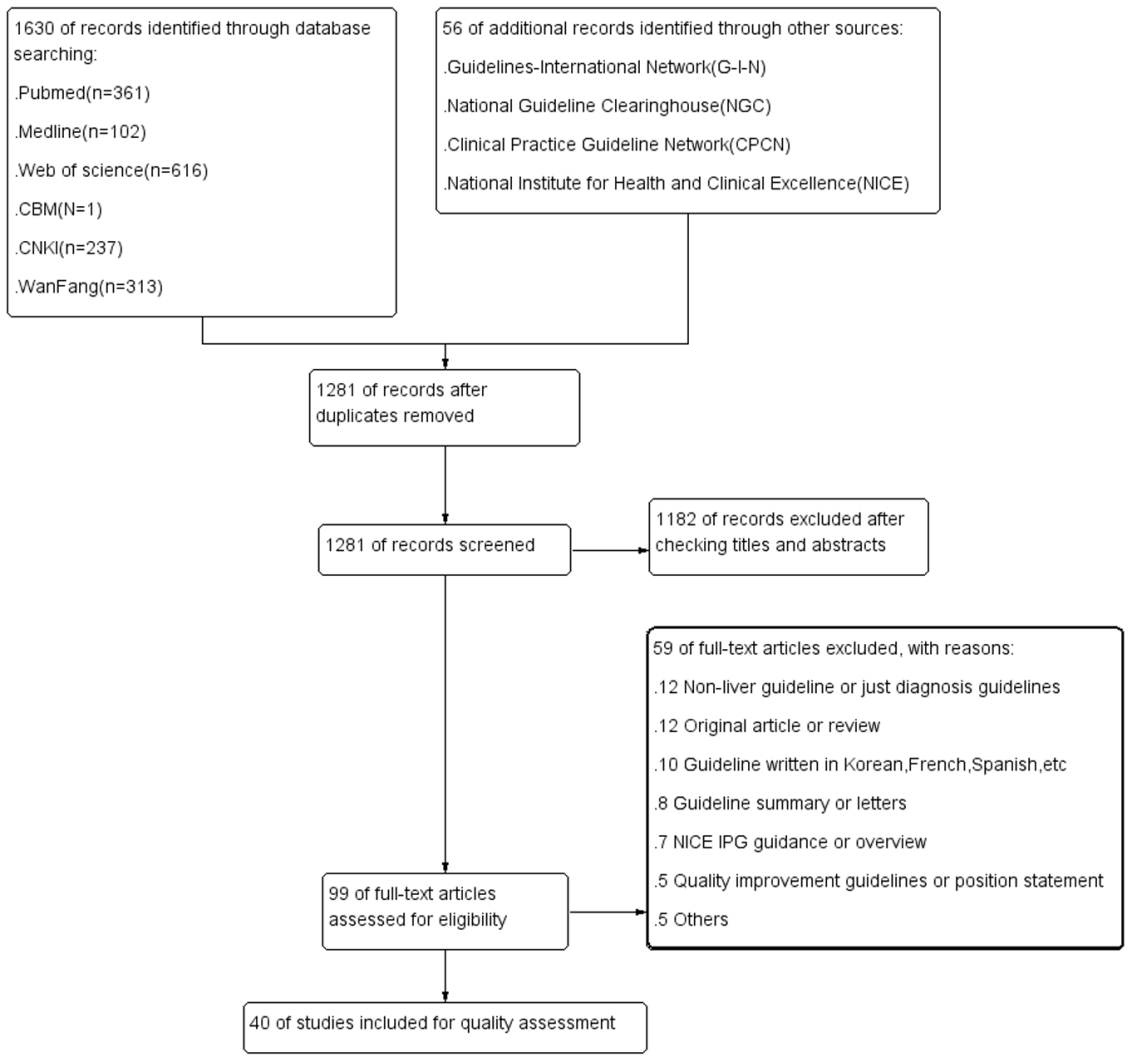
PRISMA flowchart of searching and selecting guidelines.

**Figure 2:**
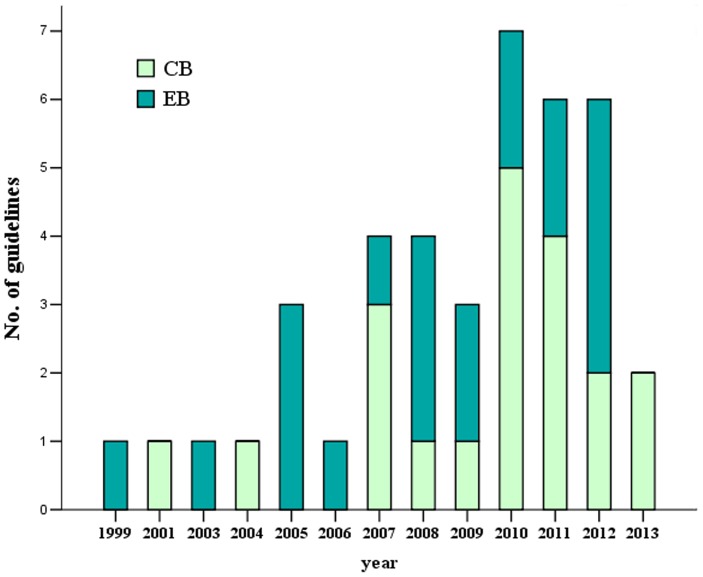
Bibliometric map of guidelines on the treatment of HCC or metastatic liver cancer.

A total of 1,686 records were obtained after systematically searching the database and relevant websites. After an initial screening, 99 records of potential interest were identified. Of these, 59 were removed after viewing the full texts for the following reasons: a) Twelve were guidelines for non-HCC or only for diagnosis of HCC; b) Twelve were primary studies or systematic reviews; c) Ten were guidelines written in French, Korean, Spanish, etc; d) Eight were guideline summaries or letters; e) Seven were quality improvement guidelines or position statements; and f) Five were NICE IPGs or overviews. Finally, 40 guidelines published between 1999 and 2013 were included, of which 20 were evidence-based [13]–[32] and 20 were consensus-based [33]–[52] (see Figure 1 and 2 for details).

The number of guidelines has risen dramatically over the years, and the proportions of consensus-based guidelines are rising in 2010 and 2011.However, evidence-based guidelines are predominant in 2012 (Figure 2).

### Baseline characteristics of included guidelines

Among the 40 guidelines, 30 (75%) were developed for HCC [13]–[29], [33]–[38], [43]–[45], [48]–[51], seven for colorectal liver metastases (CLM) [30]–[32], [42], [46], [47], [53], and four for digestive (neuro) endocrine liver metastasis [39]–[41], [49]. Twenty guidelines (50%) were evidence-based, and twenty were consensus-based. Seven guidelines were focused on a single treatment [13], [16], [19], [26], [27], [50], [52]. For instance, the guidelines established by Devlin *et al* [13] and O'Grady *et al* [17] were applicable to adults or HIV-infected patients undergoing a liver transplantation. While those developed by Knox *et al* [19], Kaneko *et al* [38], and NICE [26] were guidelines on the use of sorafenib for patients with advanced HCC, the guideline conducted by Kennedy *et al* [39] mainly recommended yttrium-90 (Y90) microsphere brachytherapy for treating malignant liver tumors. The other 33 guidelines all provided comprehensive recommendations of treatments for HCC, which are mainly liver resection, liver transplantation, ablation, transcatheter arterial chemoembolization (TACE)/transcatheter arterial embolization (TAE), systematic chemotherapy or supportive care (Table 1).

**Table 1:** General information of guidelines included in our analysis.

			Treatment strategies							
Organization, year	Cancer	Type	Liver resection	Liver transplantation	Ablative therapy	TACE/TAE	Hormonal therapy	Systematic therapy	Radiotherapy/Radioembolization	Support care
BSG,1999[13]	HCC	EB		⊚						
BSG,2003[14]	HCC	EB	⊚	⊚	⊚	⊚	⊚	⊚		
AASLD,2005a[15]	HCC	EB	⊚	⊚	⊚	⊚	⊚	⊚	⊚	
AASLD,2005b[16]	HCC	EB		⊚						
British HIV Association,2005[17]	HCC	EB		⊚						
ESMO,2008[18]	HCC	EB	⊚	⊚	⊚	⊚		⊚		⊚
CCO,2008[19]	HCC	EB						⊚		
JMH,2008[20]	HCC	EB	⊚	⊚	⊚	⊚		⊚		⊚
AOS,2009[21]	HCC	EB	⊚	⊚	⊚	⊚		⊚	⊚	
NCCN,2009[22]	HCC	EB	⊚	⊚	⊚	⊚				⊚
ESMO,2010[23]	HCC	EB	⊚	⊚	⊚			⊚	⊚	⊚
APASL,2010[24]	HCC	EB	⊚	⊚	⊚	⊚		⊚		
AASLD,2011[25]	HCC	EB	⊚	⊚	⊚	⊚		⊚	⊚	
NICE,2012[26]	HCC	EB						⊚		
AAGH,2012[27]	HCC	EB				⊚				
EASL-EORTC,2012[28]	HCC	EB	⊚	⊚	⊚	⊚	⊚	⊚		
SASLT/SOS,2012[29]	HCC	EB	⊚	⊚	⊚	⊚		⊚	⊚	⊚
AUGS, et al, 2006[30]	CLM	EB	⊚		⊚			⊚		⊚
Netherlands,2007 [31]	CLM	EB	⊚		⊚			⊚		
CMA,2011[32]	CLM	EB	⊚		⊚			⊚	⊚	⊚
EASL,2001[33]	HCC	CB	⊚	⊚	⊚	⊚	⊚	⊚		
BASL,2004[34]	HCC	CB	⊚	⊚	⊚	⊚	⊚	⊚	⊚	
JSH,2007[35]	HCC	CB	⊚	⊚	⊚	⊚		⊚		⊚
CSLCCO, et al, 2009[36]	HCC	CB	⊚	⊚	⊚	⊚			⊚	
JSH,2010a[37]	HCC	CB	⊚	⊚	⊚			⊚		
JSH,2010b[43]	HCC	CB	⊚	⊚	⊚	⊚		⊚		⊚
JSH,2010c[44]	HCC	CB	⊚	⊚	⊚	⊚		⊚		⊚
WGOGG,2010[48]	HCC	CB	⊚		⊚	⊚		⊚		⊚
JSH,2011[45]	HCC	CB	⊚	⊚	⊚	⊚		⊚		⊚
ICC,2011[50]	HCC	CB		⊚						
France,2011[51]	HCC	CB		⊚						
SGNLCT,2012[38]	HCC	CB						⊚		
EANM,2011[49]	HCC/LM	CB							⊚	
REBOC,2007[39]	LM	CB				⊚				
ENETS,2008[40]	LM	CB	⊚	⊚	⊚	⊚		⊚	⊚	
ENETS,2012[41]	LM	CB	⊚	⊚	⊚			⊚	⊚	
ICO,2007[46]	CLM	CB	⊚					⊚		⊚
CMA,2010[42]	CLM	CB	⊚		⊚			⊚	⊚	
AHPBA,2013a[47]	CLM	CB	⊚		⊚			⊚	⊚	
AHPBA,2013b[52]	CLM	CB						⊚		
Total	30(HCC) 7(CLM) 4(LM)	20(EB) 20(CB)	28	26	27	21	5	30	13	13

EB: Evidence-based. CB: Consensus-based. HCC: Hepatocellular carcinoma; CLM: Colorectal liver metastases; LM: liver metastases; HM: Hepatic Malignancy; TACE/TAE: Transarterial chemoembolization/Transarterial embolization;BSG:British Society of Gastroenterology;AASLD:American Association for the study of Liver Disease;ESMO: European Society for Medical Oncology; CCO: Cancer Care Ontario;JMH: Japanese Ministry of Health;AOS: Asian Oncology Summit;NCCN: National Comprehensive Cancer Network; APASL: Asian Pacific Association for the study of the Liver; NICE: National Institute for Health and Clinical Excellence;AAGH: Austrian Association of Gastroenterology and Hepatology;EASL-EORTC: European Association for Study of Liver—European Organization for Research and Treatment of Cancer;SASLT/SOS: Saudi Association for the Study of Liver diseases and Transplantation/Saudi Oncology Society;AUGS: Association of Upper Gastrointestinal Surgeons;CMA: Chinese Medical Association;BASL: Belgian Association for the study of the Liver;JSH: Japan Society of Hepatology;CSLCCO: Chinese Societies of Liver Cancer and Clinical Oncology;WGOGG: World Gastroenterology Organization Global Guideline;ICC: International Consensus Conference;SGNLCT: Study Group on New Liver Cancer Therapies; EANM, European Association of Nuclear Medicine;REBOC: Radioembolization Brachytherapy Oncology Consortium;ENETS: European Neuroendocrine Tumor Society;ICO: Institute of Catalan Oncology.AHPBA:Americas Hepatopancreato-Biliary Association,

### Appraisal of guidelines

Guideline evaluation results using the AGREE II instrument are detailed in Table 2. Three appraisers independently evaluated these guidelines with a mean Kendall's coefficient of concordance of 0.935 (95% CI, 0.928 to 0. 941), which indicates a high level of reliability among evaluators.

**Table 2:** Assessment of hepatocellular and metastatic liver cancer guidelines with the AGREE II instrument.

			Domain score in %						
Organization, Reference	Type	Kendall's coefficient of concordance (W)	Scope and purpose	Stakeholder involvement	Rigor of development	Clarity of presentation	Applicability	Editorial independence	Overall recommendation*
JMH,2008[20]	EB	0.933	100	93	90	94	64	25	Strongly
AASLD,2011[25]	EB	0.931	87	61	74	98	11	86	Strongly
EASL-EORTC,2012[28]	EB	0.925	94	94	72	100	28	50	Strongly
AUGS, et al, 2006[30]	EB	0.935	96	70	81	100	29	86	Strongly
Netherlands,2007[31]	EB	0.935	89	63	86	96	71	14	Strongly
BSG,1999[13]	EB	0.953	96	83	40	100	6	14	Weakly
BSG,2003[14]	EB	0.950	96	87	63	96	6	17	Weakly
AASLD,2005a[15]	EB	0.949	91	80	47	98	21	44	Weakly
AASLD,2005b[16]	EB	0.938	80	74	63	96	21	19	Weakly
British HIV Association, 2005[17]	EB	0.892	83	59	31	96	43	33	Weakly
ESMO,2008[18]	EB	0.930	81	33	40	87	1	50	Weakly
CCO,2008[19]	EB	0.939	87	57	56	61	6	100	Weakly
AOS,2009[21]	EB	0.932	85	46	56	93	39	53	Weakly
NCCN,2009[22]	EB	0.924	69	43	36	69	39	89	Weakly
ESMO,2010[23]	EB	0.931	76	39	45	87	0	50	Weakly
APASL,2010[24]	EB	0.929	74	50	49	100	1	0	Weakly
NICE,2012[26]	EB	0.905	74	59	59	93	76	39	Weakly
AAGH,2012[27]	EB	0.934	85	52	43	85	0	50	Weakly
SASLT/SOS,2012[29]	EB	0.942	89	54	67	96	6	3	Weakly
CMA,2011[32]	EB	0.962	80	33	19	63	6	47	Weakly
JSH,2010a[37]	CB	0.951	81	54	46	91	8	6	Weakly
JSH,2010b[43]	CB	0.957	78	59	3	76	7	86	Weakly
JSH,2010c[44]	CB	0.962	85	56	5	78	6	69	Weakly
JSH,2011[45]	CB	0.971	87	67	10	74	14	78	Weakly
SGNLCT,2012[38]	CB	0.922	83	80	46	85	0	6	Weakly
REBOC,2007[39]	CB	0.879	78	80	58	85	53	89	Weakly
JSH,2007[35]	CB	0.944	87	59	30	78	11	61	Weakly
CMA,2010[42]	CB	0.952	83	70	42	87	3	22	Weakly
ICC,2011[50]	CB	0.960	81	37	22	69	6	58	Weakly
France,2011[51]	CB	0.925	83	33	10	52	6	50	Weakly
AHPBA,2013a[47]	CB	0.948	85	33	9	70	3	56	Weakly
AHPBA,2013b[52]	CB	0.923	76	37	10	59	7	56	Weakly
EASL,2001[33]	CB	0.945	87	80	22	78	7	14	Not Recommend
BASL,2004[34]	CB	0.942	76	69	21	83	7	11	Not Recommend
CSLCCO, et al,2009[36]	CB	0.943	85	76	20	81	18	22	Not Recommend
ENETS,2008[40]	CB	0.942	83	43	16	81	3	3	Not Recommend
ENETS,2012[41]	CB	0.925	72	50	21	80	6	0	Not Recommend
ICO,2007[46]	CB	0.918	83	33	16	20	6	6	Not Recommend
WGOGG,2010[48]	CB	0.898	70	33	8	30	7	6	Not Recommend
EANM,2011[49]	CB	0.910	83	37	8	9	7	6	Not Recommend
Mean (95%CI)		0.935(0.928–0.941)	83(81–86)	58(52–64)	39(31–46)	79(73–86)	16(10–23)	39(30–49)	

*The overall recommendations are based on the AGREE II evaluations according to references [9] and [10]. This recommendation should be seen as a recommendation between the currently available guidelines rather than a quality stamp of the individual guideline. Please see discussion.

Among the six domains of AGREE II, 40 guidelines were scored ≥60% with a mean of 79% to 83% for two domains, namely scope and purpose, and clarity of presentation. Sixteen guidelines were scored ≥60% for the stakeholder involvement domain and the remaining twenty-four had scores ranging from 33% to 59%. For the rigor of development domain, eight guidelines were scored ≥60% with a range of 63% to 90%, sixteen were scored 30% to 59% and the last sixteen were scored 3% to 22%. For the domain of applicability, only three guidelines were scored ≥60% with a range of 64% to 76%, and four others ranged from 39% to 53%, with 33 being scored below 30%. For the domain of editorial independence, nine guidelines were scored from 61% to 100%, and thirteen ranged from 33% to 58%, while the other eighteen were scored below 30%. Therefore, five guidelines were ‘strongly recommended’ according to AGREE II including three for HCC [20], [25], [28] and two for CLM [30], [31], and 27 additional guidelines were ‘weakly recommended’. Eight guidelines were not recommended because of poor quality [33], [34], [36], [40], [41], [46], [48], [49].

**Figure 3:**
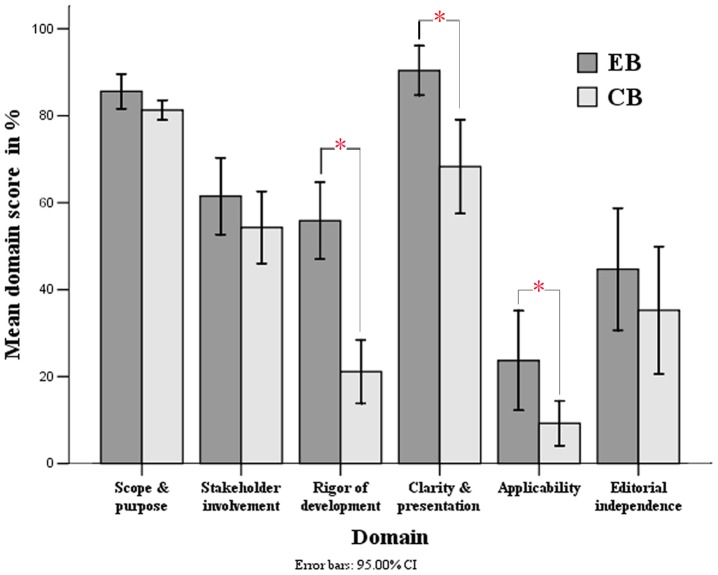
Comparison the difference between evidence-based (EB) and consensus-based (CB) guidelines in each domain. There were significantly difference between groups in domain of rigor of development, clarity & presentation and applicability with p<0.05. However, the other domains showed no significant difference between groups. *: p<0.05.

Evidence-based guidelines were superior to those established by consensus for the domains of rigor of development (p<0.001), clarity of presentation (p = 0.01), and applicability (P = 0.021). However, there was no significant difference for the other three domains (p>0.05) (Figure 3).

## Discussion

There has been a sharp increase in the number of CPGs worldwide since the 1980s [54]. As of June 2013, Guideline International Network (G-I-N) contains more than 6,400 guidelines, evidence reports and related documents (http://www.g-i-n.net/library), and the National Guideline Clearinghouse (NGC) currently includes 2,549 individual guideline summaries (http://www.guideline.gov). However, there is a great discrepancy among guidelines established by varied governments, associations, and companies or other organizations, especially with respect to their quality [6], [55], [56]. A systematic review conducted by Alonso-Coello *et al* [54] has analyzed the quality of published CPGs from 1980-2010, which showed that the quality scores measured with the AGREE instrument were moderate to low.

Zheng *et al* [57] and Chen *et al* [58] have analyzed the status of Chinese CPG development, and have concluded that considerable progress has been achieved for Chinese CPGs over time; however, all domain scores were lower than the world average, especially in rigor of development and editorial independence. There is no doubt that recommendation from low quality CPGs may mislead clinical decisions, resulting in harm to the patient. Therefore, screening for high quality CPGs is particularly vital to guide clinical practice.

In this study, it was found that the domain scores that received the highest marks as measured with AGREE II were ‘scope and purpose’ (mean 83%; 95% CI, 81% to 86%) and ‘clarity of presentation’ (mean 79%; 95% CI, 73% to 86%), which is similar to the research of Schmidt *et al* [10]. Furthermore, evidence-based guidelines are superior to consensus-based ones in terms of language, structure and layout. Because evidence-based guidelines have combined level of clinical evidence with strength of recommendations, these guidelines are more accurate and reflect a higher scientific standard.

However, there were some disappointing results regarding evidence-based guidelines in the domain of ‘stakeholder involvement’. Although the average quality score measured with AGREE II is 58%, there were 24 guidelines (60%), including eleven evidence-based guidelines that were scored less than 60%, which reflected the dearth of multidisciplinary teams and lack of accounting for views and experiences of the targeted patient population during the development of these guidelines [54]. There were various stakeholders involved, including those in steering groups, research groups involved in selecting and rating the evidence, individuals involved in formulating final recommendations, public and private funding bodies, managers, healthcare professionals, patients, employers and manufactures, but not independent individuals involved externally in reviewing the guideline[9], [59]. Their engagement of the latter group is required for various reasons such as including overlooked evidence, transparency and democracy principles, ownership, and potential policy implications [59]. Therefore, they play a vital role during guideline development, review and modification, but their involvement can also be very complex, and it needs to be inclusive, equitable, and sufficiently resourced [59].

The quality of a guideline largely depends on whether or not its methodology is rigorous and scientific. However, most guidelines received a lower score (39%) for the domain of ‘rigor of development’. Five consensus-based guidelines scored less than 30% for this domain. Although evidence-based guidelines are superior to consensus-based ones with respect to evidence gathering, quality assessment or strength of recommendations, there are still 12 evidence-based guidelines which were only scored between 30% and 60%. It is common that guidelines include references to published studies, but few of them clearly describe the searching strategy, the methodology used to formulate the final recommendations, or the dates on which guidelines were updated [10]. One reason may be the lack of methodological experts in guideline developing teams, the lack of resources needed to search for high-quality systematic reviews, or the poor reported quality of guidelines [54].

The domain of applicability mainly evaluates implementation barriers, cost factors, and monitoring criteria [9]. However, most guidelines included in this study neither discussed this field nor highlighted the tools required for facilitating or promoting guidelines, resulting in the lowest average domain scores (16%), especially for 15 evidence-based guidelines, which were scored less than 30%.

Similarly, the domain of editorial independence addresses whether the recommendations are impacted by the funding body and conflict of interests (COIs) issues which may arise from within the guideline-developing organization [9]. Potential COIs may greatly impact the content of guidelines and the recommendations. COIs was highly prevalent (150/288, 52%) among guidelines established by Canadian specialty and US specialty societies, but a large proportion of guidelines did not publicly disclose COIs [60]. A study published by Choudhry *et al* [61] showed that 87% of guideline developers had some form of interaction with a pharmaceutical company, 58% of whom had received funding support to conduct their research, and 38% of whom had served as employees or consultants in the pharmaceutical industry. In our study, 20 (50%) guidelines did not publicly disclose COIs, and 18 (45%), including seven evidence-based guidelines were scored less than 30% for this domain. Three of the five guidelines that we ‘strongly recommend’ all reported the COIs of authors in detail. In the EASL-EORTC guideline, the authors have reported the COIs at the end of guideline, however, number of affiliated authors have received research support and/or lecture fees and/or took part in clinical trials for Bayer (a pharmaceutical company)[28], which may lead certain bias for the independence of their recommendations and reliability of guideline to some extent. Therefore, recommendations based on the AGREE II ‘strongly recommend’ guidelines still need to be revised and updated according to the conclusions of properly conducted systematic reviews.

We based our recommendations of the guidelines on the AGREE II instrument as previously described [9], [10]. However, we would like to question the validity of this approach. First, such recommendations may lead clinicians to depend too much on and believe in the individual recommendations of guidelines that have achieved ‘strongly recommend’. Second, such recommendation may falsely overrate the evidence because the bar is set too low according to our experience. In short, even the ‘strongly recommend’ guidelines are not sufficiently evidence based. Thirdly, we lack evidence of any patient benefits by adopting such coarse recommendations. Therefore, the recommendations should be seen as a consequence of adopting the AGREE II methodology rather than a quality stamp on some of the guidelines as being of high methodological quality. If it is a quality stamp, it is relative to the guidelines that achieved lower ratings.

The ultimate goal of the present guideline evaluation is to recognize the faults of existing guidelines so that the necessary steps are taken to improve their quality. We found that most authors had increasingly emphasized evidence gathering and synthesis, and formulated the final recommendations when they developed their guidelines. The evidence-based guideline has become a mainstream for high quality guideline development. However, the transparency of guidelines in aspects of quality appraisal of evidence, formulation of recommendations, and the COI of authors are still insufficient, and this has become a prominent problem affecting the quality of guidelines. Some guidelines have simply classified evidence according to the study design, ignoring quality assessment of evidence, therefore making it difficult to know on which one or type of specific evidence the recommendation was based.

Although some guidelines use GRADE (the Grading of Recommendations Assessment, Development and Evaluation) as a tool for evaluating the quality of evidence and formulating the final recommendations, GRADE evidence profiles and summary of finding (SoF) tables were not presented or linked in the guidelines. Therefore, the GRADE working group has suggested that the guideline-developing committee should summarize evidence in simple, transparent and informative SoF tables and evidence profiles that provide detailed information about the reason for the quality of evidence rating [62].

Before developing a guideline, it is necessary to limit funding sources coming from industries or other institutions, or provide a formal process for discussion and public disclosure of financial COIs for authors [61], [63], [64]. When developing or updating guidelines, the AGREE II instrument is a tool that provides the methodological strategy and standard procedure [9]. When considering guideline recommendations, however, high-quality evidence (i.e., RCTs) should not always be blindly pursued [53]. Patient and societal values or preferences should be considered and incorporated with the evidence to formulate final recommendations [53], [62].

### Limitations

The study is based on published guidelines in Chinese and English journals. However, most institutions have local guidelines or rely on national guidelines (i.e., those published in books, pamphlets and government documents), none of which is published. Thus the quality of guidelines used in most clinical settings might be of lower quality than published guidelines, hence causing some degree of selection bias. The AGREE II tool mainly focuses on methodology and quality of reporting, but not on the nature of the supporting evidence. Therefore, the quality of evidence on which the recommendations are based in the ‘strongly recommended’ guidelines still needs to be systematically reviewed and amended accordingly.

## Conclusion

Although much progress has been achieved with respect to the quality of HCC and metastatic liver cancer guidelines, the overall methodological quality is moderate with poor applicability and potential conflict of interests (COIs). The evidence-based guidelines has become mainstream for high quality guideline development, such as the Japanese Ministry of Health (JMH) guideline, American Association for the Study of Liver Disease (AASLD), and European Association for the Study of Liver/European Organization for Research and Treatment of Cancer (EASL-EORTC) guideline; however, there is still a need to further increase transparency, quality of evidence rating, and the recommendation process and to address COIs issues.

## Supporting Information

Checklist S1 (DOCX)
